# Nocturnal enuresis in adults with obstructive sleep apnea hypopnea syndrome: A case report and a literature review

**DOI:** 10.1097/MD.0000000000046226

**Published:** 2025-11-28

**Authors:** Pinyi Zhou, Hongyan Li, Huijie Tang, Hongmei Li, Xiuhua Chen, Yun Fu, Daijin Huang, Jingman Qiu, Yunhui Lv

**Affiliations:** a Department of Sleep Medicine, the Affiliated Hospital of Kunming University of Science and Technology, the First People’s Hospital of Yunnan Province, Kunming, China; b Department of Neurology, Yan’an Hospital Affiliated to Kunming Medical University, Kunming, China.

**Keywords:** case report, clinical characteristics, mechanisms, nocturnal enuresis, obstructive, sleep apnea

## Abstract

**Rationale:**

To delineate the clinical phenotype, putative mechanisms, and treatment outcomes of adult-onset nocturnal enuresis attributable to obstructive sleep apnea–hypopnea syndrome (OSAHS).

**Patient concerns:**

Four consecutive obese males (aged 27–40 years) presented with nocturnal enuresis or sleep-disordered breathing; the onset of bed-wetting coincided with worsening symptoms of OSAHS. Two patients also experienced nocturnal seizure convulsions, and one had concomitant fecal incontinence.

**Diagnoses:**

Full-night polysomnography revealed extremely severe OSAHS (apnea–hypopnea index 71.2–128.8 events/h) with profound nocturnal hypoxemia (nadir SpO_2_ 27%–48%). Extensive urological, neurological, and metabolic workups disclosed no primary bladder or systemic pathology; bed-wetting was thus deemed secondary to OSAHS.

**Interventions:**

All patients were treated with noninvasive positive-pressure ventilation (continuous or bilevel positive airway pressure) and received structured weight-reduction counseling.

**Outcomes:**

Noninvasive positive-pressure ventilation produced complete remission or >90% reduction of enuretic episodes in all subjects, accompanied by marked improvement in respiratory symptoms and nocturnal oxygenation.

**Lessons:**

Adult-onset nocturnal enuresis may signal extremely severe, hypoxemia-predominant OSAHS, particularly in obese men. If no urological pathology is identified, evaluation for sleep-disordered breathing should be initiated. Prompt and effective treatment of OSAHS can significantly improve nocturnal enuresis.

## 1. Introduction

Obstructive sleep apnea–hypopnea syndrome (OSAHS) is characterized by recurrent partial or complete collapse of the upper airway during sleep, resulting in intermittent reductions or cessation of airflow.^[1]^ An estimated 936 million individuals aged 30–69 years worldwide meet diagnostic criteria for OSAHS.^[2]^ In children, the disorder affects 1–4% of the population^[3]^; prematurity further increases both prevalence and severity.^[4]^ Unlike adults, children rarely report daytime sleepiness. Instead, they typically present with hyperactivity, inattention, mood lability, and unusual sleeping postures – nonspecific manifestations that frequently lead to under-recognition.^[5]^ Because adenotonsillar hypertrophy is the predominant pediatric etiology, adenotonsillectomy remains first-line therapy.^[5,6]^ In adults, obesity, advancing age, and age-related airway remodeling are the principal risk factors; consequently, continuous positive airway pressure (CPAP) is the treatment of choice.^[1,7]^ Across all age groups, OSAHS elicits sympathetic surges, systemic inflammation, and oxidative stress that can impair growth and damage multiple organ systems.^[1,3,7]^

Nocturnal enuresis has emerged as an under-recognized complication of OSAHS. Defined as involuntary voiding during sleep, enuresis is uncommon in adults. Primary enuresis, present since infancy, is usually attributed to genetic factors, deep sleep, or psychosocial influences, whereas secondary enuresis arises after a period of established continence and necessitates evaluation for neurologic, urologic, or endocrine pathology.^[8,9]^ Among children with OSAHS, nocturnal enuresis is common (10–40%)^[10]^; however, adult data are limited, and mechanistic insights remain elusive. Case reports document resolution or substantial improvement of enuresis in adults treated with CPAP, surgery, or oral appliances^[11–15]^; nevertheless, the clinical phenotype remains poorly characterized, predisposing to misdiagnosis. In this report, we present illustrative cases and integrate current evidence to delineate the presentation, pathophysiology, and management of OSAHS-associated enuresis in adults.

## 2. Case data

### 2.1. Case 1

A 27-year-old man was admitted on March 01, 2024 with a 3-year history of snoring that had intensified over the past 12 months and was now accompanied by witnessed apneas. He had gained 40 kg during the preceding year (body mass index, BMI, 36.2 kg/m^2^) and reported prominent daytime sleepiness. One week before admission he developed nocturnal enuresis occurring once or twice nightly and a single episode of fecal incontinence on the night prior to presentation. Past medical history and physical examination were unremarkable. Brain magnetic resonance imaging (MRI) and renal ultrasonography were normal; echocardiography estimated right ventricular systolic pressure at 41 mm Hg. Polysomnography revealed an apnea–hypopnea index (AHI) of 104.2 events/h, mean oxygen saturation 61%, and nadir oxygen saturation 30%. Manual titration identified an optimal CPAP of 15 cmH₂O with 2 L/min supplemental oxygen. After 6 months of nightly therapy, the patient reported complete resolution of enuresis, reduced nocturia, uninterrupted nocturnal sleep, marked improvement in daytime alertness, and high satisfaction with the outcome.

### 2.2. Case 2

A 40-year-old man was admitted on March 16, 2024 with a 30-year history of loud snoring that had recently escalated to include nocturnal convulsions and enuresis. He had gained 3 kg over the preceding week (BMI 30.4 kg/m^2^). Family members witnessed 2 episodes characterized by apnea, repetitive blinking, and generalized limb jerking lasting 2 to 3 minutes; the patient remained unresponsive during these events and had no recall afterward. Medical history was notable for hypertension controlled with amlodipine for 8 years. Ambulatory electroencephalography and brain MRI were normal. Polysomnography demonstrated an AHI of 95.2 events/h, mean oxygen saturation 73.5%, and nadir oxygen saturation 35%. Manual titration established an optimal CPAP of 8 cmH₂O, initiated the same night. Enuresis and nocturnal motor spells ceased immediately. At the 3-month follow-up, the patient remained dry, blood pressure was stable, daytime vigilance had normalized, and he reported satisfactory work and daily function.

### 2.3. Case 3

A 38-year-old man (BMI 28.6 kg/m²) was admitted on April 08, 2024 with a 10-day history of nightly enuresis accompanied by loud snoring, chest tightness, palpitations, and arousals with shortness of breath; daytime sleepiness was prominent, yet diurnal micturition was normal. Past medical history and physical examination were unremarkable. Laboratory studies revealed polycythemia (hemoglobin 204 g/L) and room-air arterial blood gas values of PaO₂ 59 mm Hg and PaCO₂ 45 mm Hg. Renal ultrasonography showed a tiny calculus in the upper pole of the left kidney and increased prostatic echogenicity; brain MRI, ambulatory electroencephalography, and echocardiography were normal. Polysomnography documented an AHI of 71.2 events/h, mean oxygen saturation 76%, and nadir oxygen saturation 27%. Manual titration established optimal bilevel positive-airway-pressure support in spontaneous/timed mode (EPAP 10 cmH₂O, IPAP 22 cmH₂O) with 2 L/min supplemental oxygen. After 1 month of nightly therapy, the patient achieved complete resolution of enuresis and daytime sleepiness, and he expressed high satisfaction with the outcome.

### 2.4. Case 4

A 37-year-old man presented to the emergency department on February 05, 2025 with a 1-week history of orthopnea and paroxysmal nocturnal dyspnea. During the same period, he experienced nightly enuresis and 5 witnessed nocturnal convulsions, along with daytime urge incontinence. Body weight had increased 10 kg over the preceding year (BMI 37.9 kg/m²). He had snored loudly for at least 15 years. Renal ultrasonography revealed a simple upper-pole cyst and a small middle-calyx calculus in the right kidney; non-contrast head computed tomography and echocardiography were normal. Polysomnography disclosed an AHI of 128.8 events/h, mean oxygen saturation 88%, and nadir oxygen saturation 48%. Manual titration identified an optimal CPAP of 13 cmH₂O. After 3 months of compliant therapy, the patient’s respiratory symptoms, enuresis, and nocturnal motor events had completely resolved; he considered the result excellent.

## 3. Literature review

A systematic search of Web of Science, PubMed, and Scopus through May 2025 using the terms “obstructive sleep apnea,” “OSA,” “OSAHS,” and “nocturnal enuresis” yielded 67 English-language citations. After exclusion of studies focusing on childhood enuresis, adult daytime incontinence, and duplicate records, 7 publications remained (Table 1): one cohort study comprising 23 women and 6 case series or single-case reports. Collectively, these reports describe 39 adults (12 men, 27 women; age range 26–68 years) with coexisting OSAHS and nocturnal enuresis.

**Table 1:** Literature search results for adult OSAHS with nocturnal enuresis.

Literature reports	Year	Type	Cases	Male/Female	Age (yr)	BMI (kg/m^2^)	AHI (n)	Nadir O_2_ %	PAP and other treatments and outcomes
Brown et al^[11]^	1995	Case Report	1	1/0	26	37.3	–	46	Enuresis symptoms improved following CPAP treatment.
Everaert et al^[12]^	1995	Case Report	1	1/0	35	–	36	–	Enuresis symptoms improved following CPAP treatment.
Yokoyama et al^[16]^	1995	Case Report	1	0/1	53	34.6	–	36	After treatment with imipramine hydrochloride and acetazolamide, apneas decreased and enuresis improved. Following a weight loss of 10 kg, medications were discontinued, and enuresis did not recur.
Ulfberg and Thuman^[13]^	1996	Case Report	3	3/0	50–68	–	–	30–70	Enuresis symptoms improved following CPAP treatment; in one case, due to intolerance of CPAP, enuresis symptoms improved following treatment with an oral appliance.
Kramer et al^[14]^	1998	Case Report	5	5/0	29–46	35.2–46.7	80–126	66–82	Enuresis symptoms improved following CPAP treatment.
McInnis et al^[15]^	2017	Case Report	5	2/3	27–59	31–60.3	7.21–125.3	50	Enuresis symptoms improved following CPAP treatment.
Myer et al^[17]^	2020	Original Article	23	0/23	–	–	–	–	Among 46 women at high risk for OSAHS, 23 exhibited symptoms of nocturnal enuresis.

“–” indicates not specified in the literature.

AHI = apnea–hypopnea index, BMI = body mass index, CPAP = continuous PAP, OSAHS = obstructive sleep apnea–hypopnea syndrome, PAP = positive airway pressure.

Ulfberg and Thuman^[13]^ reported three obese men whose enuresis persisted after transurethral prostatectomy; complete resolution of enuresis was achieved following adequate control of OSAHS with continuous positive airway pressure (CPAP) or an oral appliance. Kramer et al^[14]^ documented 5 morbidly obese men with severe OSAHS, excessive daytime sleepiness, and nightly enuresis; CPAP therapy resulted in complete cessation of enuresis. Brown et al^[11]^ described a 34-year-old man in whom rapid weight gain preceded new-onset enuresis; CPAP reversed both sleep-disordered breathing and nocturnal enuresis. Everaert et al^[12]^ evaluated a 42-year-old man whose normal urodynamic findings and voiding cystourethrogram contrasted with marked obesity, hypersomnolence, and an AHI > 60 events/h; enuresis resolved on the first night of CPAP. McInnis et al^[15]^ followed 5 obese adults (1 with mild and 4 with severe OSAHS) whose enuresis and daytime sleepiness resolved with nasal CPAP. Yokoyama et al^[16]^ simultaneously recorded polysomnography and intravesical pressure in a 53-year-old woman, demonstrating that each obstructive apnea produced an abrupt increase in intra-abdominal pressure and a detrusor spike >60 cmH₂O, sufficient to overcome urethral closure pressure. Treatment with imipramine plus acetazolamide reduced respiratory events and eliminated enuresis. Finally, Myer et al^[17]^ surveyed urogynecology clinic patients and found that 38.5% (n = 46) screened high-risk for OSAHS; these women reported significantly more frequent and more severe nocturnal enuresis than low-risk controls (*P* < .001), suggesting that OSAHS is an under-recognized contributor to adult nocturnal enuresis.

## 4. Discussion

Lower urinary tract symptoms are a well-documented comorbidity of OSAHS and are classified into storage, voiding, and post-micturition complaints. Storage symptoms – urinary frequency, urgency, incontinence, and nocturia – are the predominant urological manifestations of OSAHS and significantly impair quality of life.^[18]^ A cross-sectional study of 1236 men showed that the prevalence of urinary incontinence and nocturia among adults with OSAHS was 3.32% and 2.02%, respectively – figures significantly higher than in men without OSAHS.^[19]^ Nocturnal enuresis, regarded as sleep-related incontinence, has a multifactorial and incompletely understood pathophysiology; it is most common in female children.^[9]^ In adults, nocturnal enuresis is uncommon, with an estimated prevalence of 2–3%,^[20]^ and its sex distribution remains controversial.^[21,22]^ The association between OSAHS and nocturnal enuresis is well established in children,^[10]^ but data in adults are sparse, and reliable epidemiological estimates are lacking. Notably, a questionnaire-based study in postmenopausal women at high risk for OSAHS reported an nocturnal enuresis prevalence of 46%, suggesting that the link may be particularly pronounced in specific populations.^[23]^

The present report describes 4 obese men with severe OSAHS and profound nocturnal hypoxemia in whom nocturnal enuresis developed concomitantly with worsening of sleep-disordered breathing. In all patients, nocturnal enuresis resolved or improved markedly on the first night of noninvasive positive-pressure ventilation after polysomnographic confirmation of OSAHS – an observation consistent with previous case series^[11–15]^ and indicative of a causal relationship.

The mechanisms by which OSAHS may precipitate adult nocturnal enuresis are likely multifactorial. Nocturnal polyuria, detrusor overactivity during sleep, and an elevated arousal threshold are the three principal contributors to nocturnal enuresis and have been attributed to subtle brain-stem dysfunction.^[20]^

(1) OSAHS is significantly associated with nocturnal polyuria. In individuals ≥45 years, the prevalence of nocturia among OSAHS patients approaches 44–68%.^[24]^ A meta-analysis further demonstrated a dose–response relationship between OSAHS severity and nocturia frequency,^[25]^ indicating that progressive worsening of sleep-disordered breathing confers incremental risk for nighttime voiding. The underlying mechanism centers on dysregulated secretion of atrial natriuretic peptide (ANP).^[26]^ ANP, synthesized and released by atrial myocytes, antagonizes the renin–angiotensin–aldosterone axis and sympathetic outflow while promoting renal sodium and water excretion.^[27]^ In OSAHS, nocturnal increases in intrathoracic negative pressure, intermittent hypoxia, hypercapnia, and sleep fragmentation evoke surges in sympathetic activity and systemic/pulmonary vasoconstriction, thereby augmenting cardiac preload. The resulting atrial stretch precipitates exaggerated ANP release and concomitant suppression of antidiuretic hormone, collectively enhancing natriuresis and diuresis.^[28]^ Untreated OSAHS is characterized by elevated nocturnal ANP levels that fall precipitously after effective therapy.^[29,30]^

(2) Micturition is a highly coordinated reflex requiring synergistic interaction between the detrusor, internal urethral sphincter, and supraspinal volitional control. Detrusor overactivity – defined as involuntary phasic contractions during the storage phase – produces rises in intravesical pressure and gives rise to urgency, frequency, and urge incontinence. Both detrusor overactivity and the broader clinical entity of overactive bladder are regarded as central mechanisms underlying enuresis in children with OSAHS^[10]^ and have been documented in adults with the disorder.^[31]^ Proposed pathways include intermittent hypoxia-induced oxidative stress injury; animal studies demonstrate that chronic intermittent hypoxia disrupts bladder ultrastructure, yielding detrusor dys-coordination, reduced compliance, and enhanced spontaneous contractile activity.^[26]^ Conversely, urodynamic investigation of an adult with OSAHS-related enuresis revealed neither daytime nor nighttime detrusor overactivity,^[16]^ underscoring the potential heterogeneity of pathophysiologic mechanisms. Additional controlled studies are warranted to clarify the contribution of detrusor instability to OSAHS-associated nocturnal enuresis.

(3) Upper-airway obstruction and sustained nocturnal hypoxemia in OSAHS fragment sleep and evoke recurrent cortical arousals. Over time, this repetitive disruption can elevate the arousal threshold, a phenomenon documented in 30–50% of patients with OSAHS.^[28]^ While low arousal thresholds are frequently cited as drivers of premature awakening that blunt the accumulation of physiologic stimuli (e.g., hypercapnia and negative pharyngeal pressure), thereby diminishing pharyngeal dilator recruitment and worsening OSAHS severity,^[32–34]^ high arousal thresholds are an under-appreciated correlate of disease burden. Higher thresholds, more common in severe OSAHS, predispose to prolonged obstructive events, deeper desaturation, and pronounced hypercapnia.^[35,36]^ Such blunted responsiveness is also associated with systemic sequelae, including hypertension and metabolic syndrome.^[36,37]^ Importantly, an elevated arousal threshold reduces perception of bladder distension or detrusor contractions, increasing the likelihood of enuresis.^[20]^

Case observations further demonstrate that abrupt rises in intra-esophageal and intra-abdominal pressure during obstructive respiratory events can be transmitted to the bladder, producing intravesical pressures that exceed the maximal urethral closure pressure (≈60 cmH₂O) and precipitate involuntary voiding.^[16]^ Obesity – an established risk factor for both OSAHS and nocturnal enuresis^[23,38]^ – amplifies this risk by several convergent mechanisms: increased intra-abdominal load elevates baseline bladder pressure^[39]^; adiposity worsens the severity of apneic episodes and nocturnal hypoxemia; and obesity-related systemic inflammation promotes detrusor overactivity and subclinical cystitis, thereby reducing functional bladder capacity.^[10]^ Collectively, these factors heighten the probability of enuresis in patients with OSAHS.

In this case series, reversal of nocturnal hypoxemia after initiation of positive airway pressure was accompanied by immediate cessation of bed-wetting in every patient, providing circumstantial evidence for a causal link between OSAHS and nocturnal enuresis. Reports of enuresis attributable to OSAHS in adults remain limited to small case series, most likely because of the low background prevalence of the symptom and under-recognition of OSAHS in this clinical context. Our structured review of seven relevant studies identified a male predominance (male:female = 12:4) after exclusion of one investigation that enrolled only women.^[17]^ Across these reports, patients were typically obese and exhibited severe apneic events with profound nocturnal desaturation; comprehensive urological evaluation was consistently unremarkable, and correction of hypoxemia through treatment of OSAHS resulted in remission of enuresis. The present observations corroborate these earlier findings and underscore the importance of systematic OSAHS screening in any adult who presents with secondary nocturnal enuresis.

Beyond conventional noninvasive positive airway pressure therapy, alternative interventions that alleviate hypoxemia have also been shown to abolish OSAHS-related enuresis. Ulfberg and Thuman^[13]^ documented resolution of wetting with an oral appliance, whereas Yokoyama et al^[16]^ reported prompt cessation of enuresis after acetazolamide eliminated apneic episodes and hypoxemia; symptoms did not recur after weight reduction and drug discontinuation.

This case series is intended to heighten clinical awareness of the relationship between OSAHS and adult nocturnal enuresis. Early identification and treatment of OSAHS not only mitigate systemic hypoxic injury but may also reverse associated urological symptoms. Nevertheless, several questions remain unanswered. Rigorous animal studies and large-scale prospective trials are required to delineate the precise pathophysiological pathways linking intermittent nocturnal hypoxia to involuntary micturition. Long-term follow-up of enuretic patients after successful OSAHS therapy is also warranted to confirm the durability of symptom remission.

## Acknowledgments

We thank all participants and staff involved in this study.

## Author contributions

**Conceptualization:** Pinyi Zhou, Hongyan Li, Huijie Tang, Hongmei Li, Xiuhua Chen, Yun Fu, Daijin Huang, Jingman Qiu.

**Data curation:** Yun Fu.

**Funding acquisition:** Yunhui Lv.

**Investigation:** Pinyi Zhou, Hongyan Li, Daijin Huang, Jingman Qiu.

**Methodology:** Pinyi Zhou.

**Project administration:** Yunhui Lv.

**Resources:** Yunhui Lv.

**Supervision:** Huijie Tang.

**Validation:** Hongmei Li, Yun Fu, Jingman Qiu.

**Visualization:** Xiuhua Chen.

**Writing – original draft:** Pinyi Zhou, Hongyan Li, Huijie Tang, Hongmei Li, Xiuhua Chen, Yun Fu, Daijin Huang, Jingman Qiu, Yunhui Lv.

**Writing – review & editing:** Pinyi Zhou, Hongyan Li, Huijie Tang, Hongmei Li, Xiuhua Chen, Yun Fu, Daijin Huang, Jingman Qiu, Yunhui Lv.
